# Hospital-onset bacteremia in the neonatal intensive care unit: strategies for risk adjustment

**DOI:** 10.1017/ice.2024.238

**Published:** 2025-04

**Authors:** Erica C. Prochaska, Shaoming Xiao, Elizabeth Colantuoni, Nora Elhaissouni, Reese H. Clark, Julia Johnson, Sagori Mukhopadhyay, Ibukunoluwa C. Kalu, Danielle M. Zerr, Patrick J. Reich, Jessica Roberts, Dustin D. Flannery, Aaron M. Milstone, Group Information: CDC Prevention Epicenters Program

**Affiliations:** 1 Division of Pediatric Infectious Diseases, Department of Pediatrics, Johns Hopkins University School of Medicine, Baltimore, MD, USA; 2 Department of Hospital Epidemiology and Infection Control, Johns Hopkins Health System, Baltimore, MD, USA; 3 Department of Epidemiology, University of Colorado Anschutz Medical Campus, Aurora, CO, USA; 4 Department of Biostatistics, Bloomberg School of Public Health, Johns Hopkins University, Baltimore, MD, USA; 5 Pediatrix Medical Group, Sunrise, FL, USA; 6 Division of Neonatology, Department of Pediatrics, Johns Hopkins University School of Medicine, Baltimore, MD, USA; 7 Department of International Health, Johns Hopkins Bloomberg School of Public Health, Baltimore, MD, USA; 8 Division of Neonatology, Department of Pediatrics, Children’s Hospital of Philadelphia, Philadelphia, PA, USA; 9 Division of Pediatric Infectious Diseases, Department of Pediatrics, Duke University School of Medicine, Durham, NC, USA; 10 Division of Infectious Diseases, Department of Pediatrics, University of Washington and Seattle Children’s Hospital, Seattle, WA, USA; 11 Division of Pediatric Infectious Diseases, Department of Pediatrics, Washington University School of Medicine, St. Louis, MO, USA; 12 Division of Neonatology, Department of Pediatrics, Emory University School of Medicine & Children’s Healthcare of Atlanta, Atlanta, GA, USA

## Abstract

**Objective:**

To quantify the impact of patient- and unit-level risk adjustment on infant hospital-onset bacteremia (HOB) standardized infection ratio (SIR) ranking.

**Design:**

A retrospective, multicenter cohort study.

**Setting and participants:**

Infants admitted to 284 neonatal intensive care units (NICUs) in the United States between 2016 and 2021.

**Methods:**

Expected HOB rates and SIRs were calculated using four adjustment strategies: birthweight (model 1), birthweight and postnatal age (model 2), birthweight and NICU complexity (model 3), and birthweight, postnatal age, and NICU complexity (model 4). Sites were ranked according to the unadjusted HOB rate, and these rankings were compared to rankings based on the four adjusted SIR models.

**Results:**

Compared to unadjusted HOB rate ranking (smallest to largest), the number and proportion of NICUs that left the fourth quartile (worst-performing) following adjustments were as follows: adjusted for birthweight (16, 22.5%), birthweight and postnatal age (19, 26.8%), birthweight and NICU complexity (22, 31.0%), birthweight, postnatal age and NICU complexity (23, 32.4%). Comparing NICUs that moved into the better-performing quartiles after birthweight adjustment to those that remained in the better-performing quartiles regardless of adjustment, the median percentage of low birthweight infants was 17.1% (Interquartile Range (IQR): 15.8, 19.2) vs 8.7% (IQR: 4.8, 12.6); and the median percentage of infants who died was 2.2% (IQR: 1.8, 3.1) vs 0.5% (IQR: 0.01, 12.0), respectively.

**Conclusion:**

Adjusting for patient and unit-level complexity moved one-third of NICUs in the worst-performing quartile into a better-performing quartile. Risk adjustment may allow for a more accurate comparison across units with varying levels of patient acuity and complexity.

## Introduction

Central line-associated bloodstream infections (CLABSIs) are associated with increased mortality; however, these events are largely preventable within comprehensive infection prevention programs.^1,2^ Infants hospitalized in the neonatal intensive care unit (NICU) have unique risk factors for bloodstream infections, and these infections are associated with increased mortality, morbidity, and prolonged length of admission.^3–5^ The high morbidity and preventability of CLABSIs makes them an important healthcare quality measure, and the Centers for Disease Control and Prevention (CDC) monitors CLABSIs for benchmarking and quality improvement. To compare hospital CLABSI events, the CDC calculates standardized infection ratios (SIRs), which are defined as the observed number of CLABSI events in a unit or hospital divided by the expected number of CLABSI events.^6^ The expected number of CLABSI events is calculated from nationally aggregated data and is risk adjusted for patient, unit, and hospital characteristics.^7^ Neonatal CLABSI SIR also adjusts for birthweight categories, because birthweight is an established risk factor for infant CLABSI.^8,9^ The Centers for Medicare and Medicaid Services (CMS) incorporates hospital CLABSI SIRs into a hospital-acquired condition reduction program score. These scores determine which hospitals will be subject to CMS payment reductions.^10^ Despite the importance of CLABSI surveillance and reduction, specific concerns about CLABSI reporting remain, including sensitivity of the SIR to a low number of events, changing CLABSI definitions over time that complicates monitoring of trends, insufficient risk adjustment for complex patient populations, and a time-intensive adjudication process.^11,12^

To improve and automate electronic data exchange, the CDC is adopting Fast Health Interoperable Resources (FHIR), an electronic data exchange program.^13^ This program allows for automated, electronic extraction of HAI data. Using FHIR, the CDC will implement hospital-onset bacteremia or fungemia (HOB) as a new HAI measure to expand surveillance to all bloodstream infections in hospitalized patients, including those not associated with central lines.^11,14^ HOB is defined as a positive blood culture for bacteria or fungi after day 3 of hospital admission.^11^ We previously estimated the infant HOB rate to be 1.1 events per 1,000 NICU days, and 54% of events occurred in the absence of a central line.^15^ Infants who had a HOB event had a 5% increased, attributable mortality as compared to matched infants without a HOB event. We also found that birthweight <1500 g, postnatal age 4–14 days, and central line presence are risk factors for HOB among infants admitted to the NICU. In addition, infants born ≥1500 grams with postnatal age >42 days had an increased risk of hospital-onset bacteremia. HOB risk adjustment and SIR ranking models have been researched in adult patient cohorts; however, to our knowledge, there are no HOB SIR risk adjustment models using infant data.^11,14,16^ Pediatric-specific risk adjustment is crucial to creating national benchmarks and comparing hospital performance for the care of infants and children. Risk adjustment may also affect CMS payments should HOB be incorporated into future CMS scores. Our prior findings indicate that both birthweight and postnatal age might be important risk adjustment variables for infant HOB. This study’s primary objective was to measure the impact of patient- and unit-level risk adjustment on infant HOB SIR ranking.

## Methods

### Study design, setting, and population

We performed a multicenter, retrospective cohort study of 322 NICUs in the United States. Seven academically affiliated NICUs and 315 NICUs in the Pediatrix Medical Group contributed data from 2016 to 2021. Pediatrix Medical Group is a United States healthcare provider that specializes in maternal, neonatal and pediatric care. Academically affiliated NICUs extracted data from the electronic medical record and Pediatrix Medical Group provided data from their Pediatrix Clinical Data Warehouse.^17^ Data included postnatal days, birthweight, gestational age, central line presence, microbiology results, age at admission, and age at discharge, transfer, or death. Infants with missing birthweight, gestational age, sex, or age were excluded. Inborn infants and infants transferred into a participating NICU were included, regardless of postnatal age. Cultures with missing results were excluded. NICUs with more than 20% missing culture data were excluded. NICUs with predicted HOB count of <1 were excluded to be consistent with National Healthcare Safety Network (NHSN) reporting guidelines.^6^ Two hundred and seventy-seven NICUs provided antibiotic data and were included in a sub-analysis of non-commensal and treated commensal HOB events. The Johns Hopkins School of Medicine institutional review board approved the study with a waiver of consent, and the protocol was reviewed and approved by each site’s respective institutional review board.

### Definitions

The primary outcomes were unadjusted HOB rates and HOB SIRs. HOB was defined as growth of bacteria or fungi from blood culture on day ≥4 of admission to the hospital.^14,18,19^ Subsequent HOB events were included if a blood culture was positive for bacteria or fungi ≥14 days after a prior positive culture. Cultures associated with early-onset sepsis (positive blood culture in first 3 days after birth or blood culture positive for group B *Streptococcus* in first 7 days after birth) were excluded, but infants with early-onset sepsis were eligible for HOB 14 days after their prior positive culture. These early-onset sepsis definitions were based upon anticipated CDC definitions. Common commensal organisms, as defined by NHSN definitions, can cause invasive infections in preterm and critically ill infants, and therefore were included.^20,21^ A treated commensal HOB was defined as ≥5 days of continuous antibiotics started within 2 calendar days of a commensal organism growth from blood culture. The first 3 days of hospital admission and the 14 days after a prior positive blood culture were excluded from at-risk time regardless of organism. The HOB rate was expressed as the number of HOB events per 1,000 patient days. The SIR was defined as a NICU’s observed HOB events divided by the expected number of HOB events after risk adjustment obtained from models described below, with SIRs > 1 indicating a greater number of observed HOB events than expected given the characteristics of the infants at the site. NICUs were ranked (ordered from smallest to largest) based on the unadjusted HOB rate and the HOB SIRs. We defined worst-performing sites as the quartile of sites with the largest unadjusted HOB rate or largest HOB SIR, ie the fourth quartile (>75^th^ percentile). This method was used because hospitals in the >75^th^ percentile of hospital-acquired condition scores are subject to CMS payment reductions.^10^

Adjustment variables included infant birthweight, infant postnatal age, and NICU complexity. Birthweight groups were defined using NHSN CLABSI risk adjustment birthweight groups: ≤ 750 g, 751–1000 g, 1001–1500 g, 1501–2500 g, and >2500 g.^7^ To adjust for postnatal age, we included postnatal age >42 days as an interaction term with birthweight based on prior work demonstrating that infants born ≥1500 g had a relative increased risk of HOB after postnatal day 42.^15^ NICU complexity was represented with the following characteristics: mean admissions per year, percentage of low birthweight (<1500 g) infants admitted, and the percentage of infants transferred into the NICU from outside NICUs, which were treated as continuous measures and modeled using natural cubic splines. To determine the degrees of freedom (df) for natural cubic splines, we used 10-fold cross-validation and calculated the mean Akaike Information Criteria (AIC) for each df combination and selected the combination with the lowest AIC.^22^ Birthweight and postnatal age were selected as adjustment variables because we previously demonstrated that these variables have a strong independent association with HOB risk.^15^

### Data analysis

The unit of analysis is the NICU. Descriptive analyses of NICU and infant variables were performed and reported as quartiles (median, interquartile ranges) and frequency (percentages) as appropriate. First, the crude HOB rate for each NICU was computed as the number of HOB events per 1,000 patient days. Then, four models were used to estimate risk-adjusted HOB events for each NICU; model 1: CDC birthweight groups, model 2: CDC birthweight groups separately within postnatal age categories, by including a statistical interaction between birthweight and postnatal age, model 3: NICU complexity and CDC birthweight groups, and model 4: CDC birthweight group separately within postnatal age categories and NICU complexity. The expected HOB rates based on each of the four adjustments were estimated using a Poisson regression model for the number of HOB events that included patient days as offset and the respective adjustment variables. The site rankings based upon the unadjusted HOB rates were compared with the four HOB SIR rankings. Spearman correlation coefficients were calculated to compare HOB SIR ranks between models and NICU characteristics with NICU HOB rates. Analyses were performed using R Statistical Software v4.2.3 (R Core Team, 2023).

## Results

**Figure 1.**
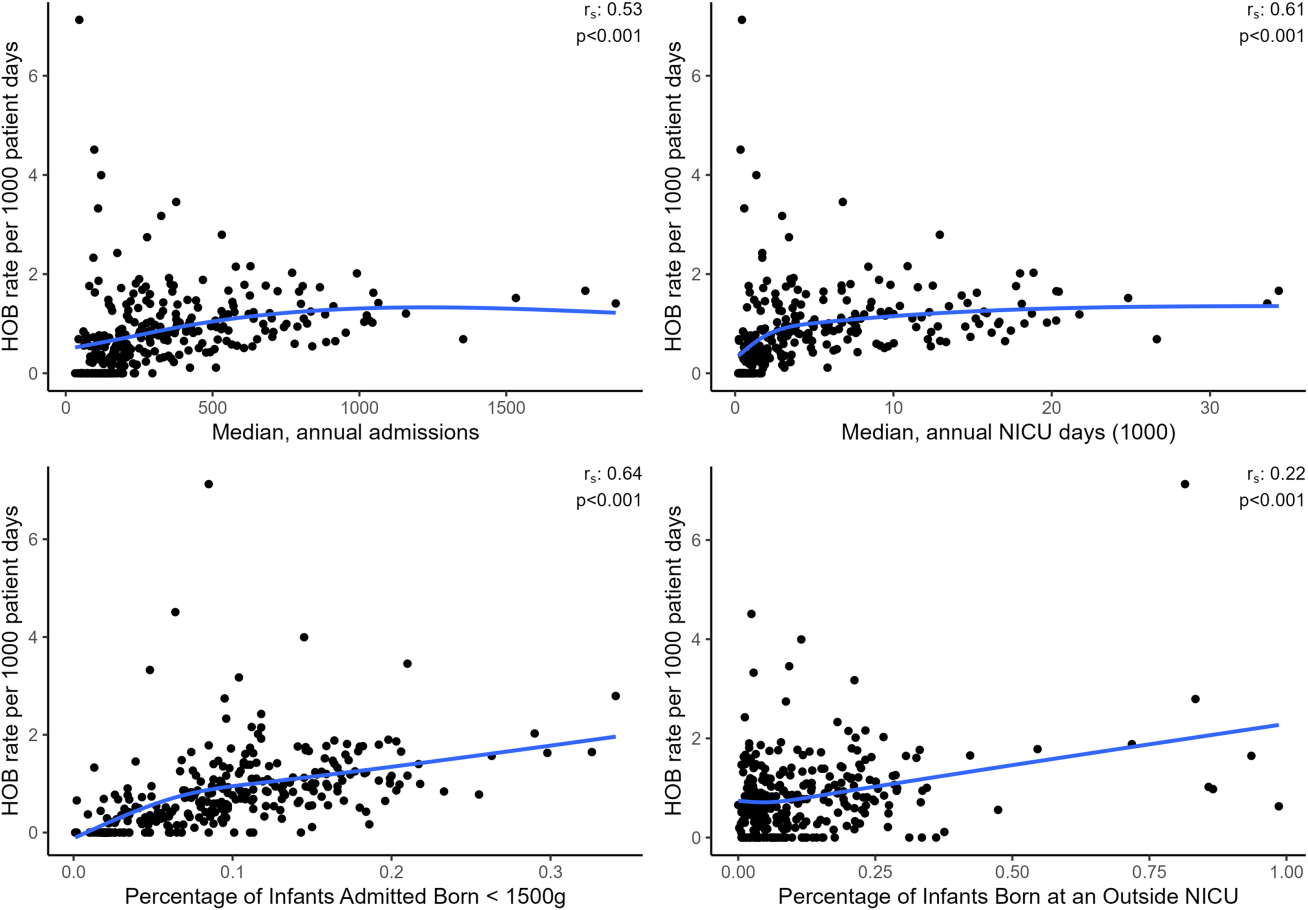
Scatter Plots of Neonatal Intensive Care Unit-level Characteristics with Site Hospital-Onset Bacteremia Rates. Correlation coefficients are shown as r_s_.

Data was obtained from 322 NICUs. A total of 284 NICUs met inclusion criteria. Nineteen NICUs were excluded due to having more than 20% missing culture data, and an additional 19 were excluded due to having an expected HOB count <1. The median proportion of missing culture data among included NICUs was 3.5% (interquartile range (IQR): 1.7%–7.1%). The mean, annual admissions per NICU ranged from 36.6 to 2028.6 with a median of 326 (IQR: 192.1–569.5). The median length of stay per NICU ranged from 2.0 to 17 days with an overall median of 8 days (IQR: 6–10). Across sites, the median percentage of infants born <1500 g was 9.4% (IQR: 5.4–13.9%). The median percentage of infants born ≥1500 g who were admitted past 42 days within all sites was 1.8% (IQR: 0.8%–3.1%). The unadjusted HOB rate ranged from 0.0 to 7.1 per 1,000 patient days, with a median of 0.7 per 1,000 patient days (IQR: 0.3–1.2). The unadjusted HOB rate was correlated with the percentage of infants born <1500 g (Spearman coefficient, 0.64, *P* value (*P*) < .001), median annual patient days (Spearman coefficient 0.61, *P* < .001), mean admissions per year (Spearman coefficient 0.53, *P* < .001), and percentage of admitted infants born at an outside NICU (Spearman coefficient, 0.22, *P* < .001) (Figure 1).

**Figure 2.**
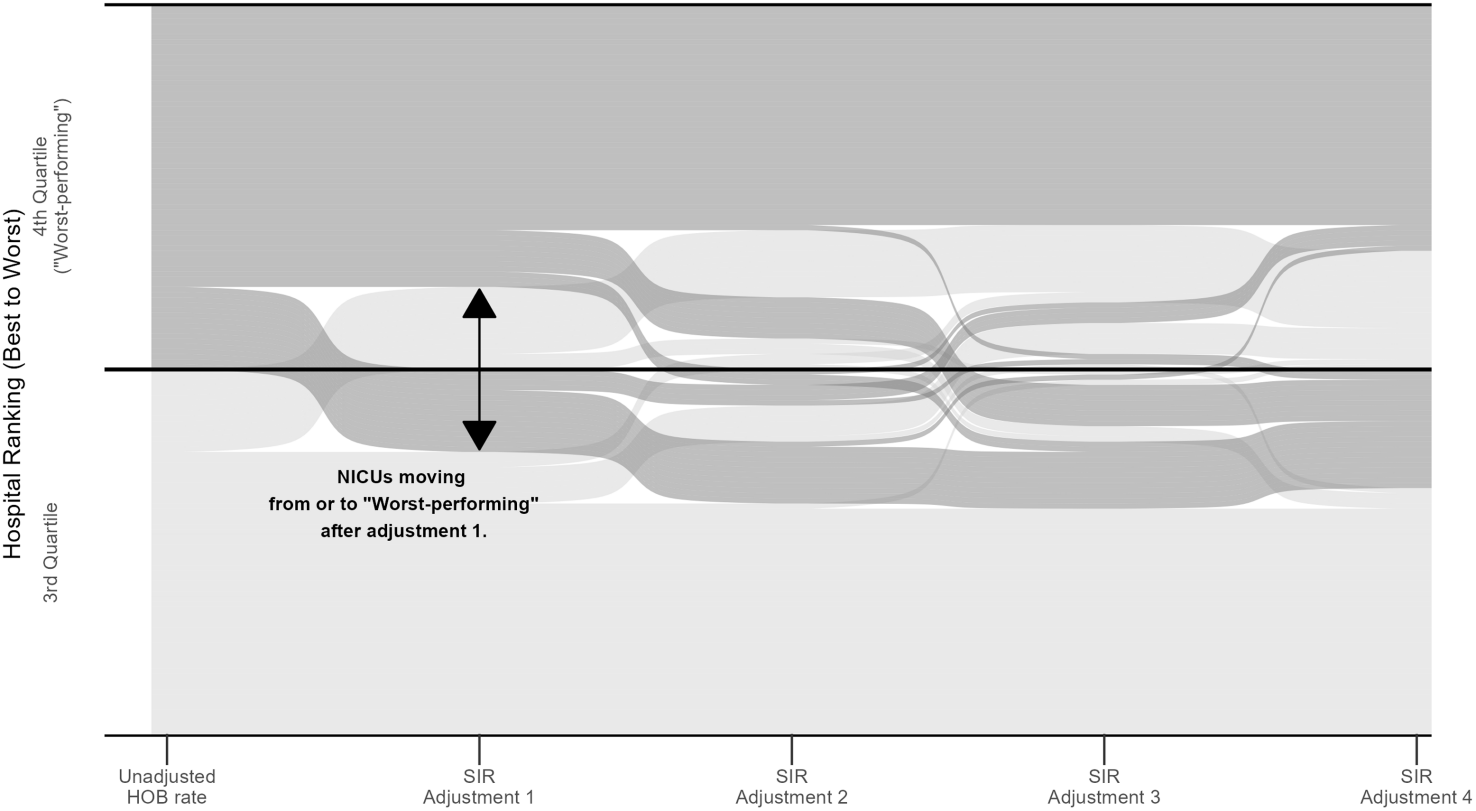
Alluvial plot of neonatal intensive care unit (NICU) rankings based upon the unadjusted HOB rate and standardized infection ratios (SIR) calculated from four adjusted models: birthweight (model 1), birthweight and postnatal age (model 2), birthweight and NICU complexity (model 3), and all variables (model 4). Based upon unadjusted HOB rate, the sites in the fourth quartile (worst-performing) are shown in dark gray and first-third quartiles (better-performing) are shown in light gray. Forty-four sites remained in the fourth quartile and 185 sites remained in the first-third quartiles regardless of adjustment. Across all adjustment strategies, 55 sites experienced a change into or out of the fourth quartile. The plot is truncated to show the 55 sites that experienced a change in performance quartiles and is not to scale.

**Figure 3.**
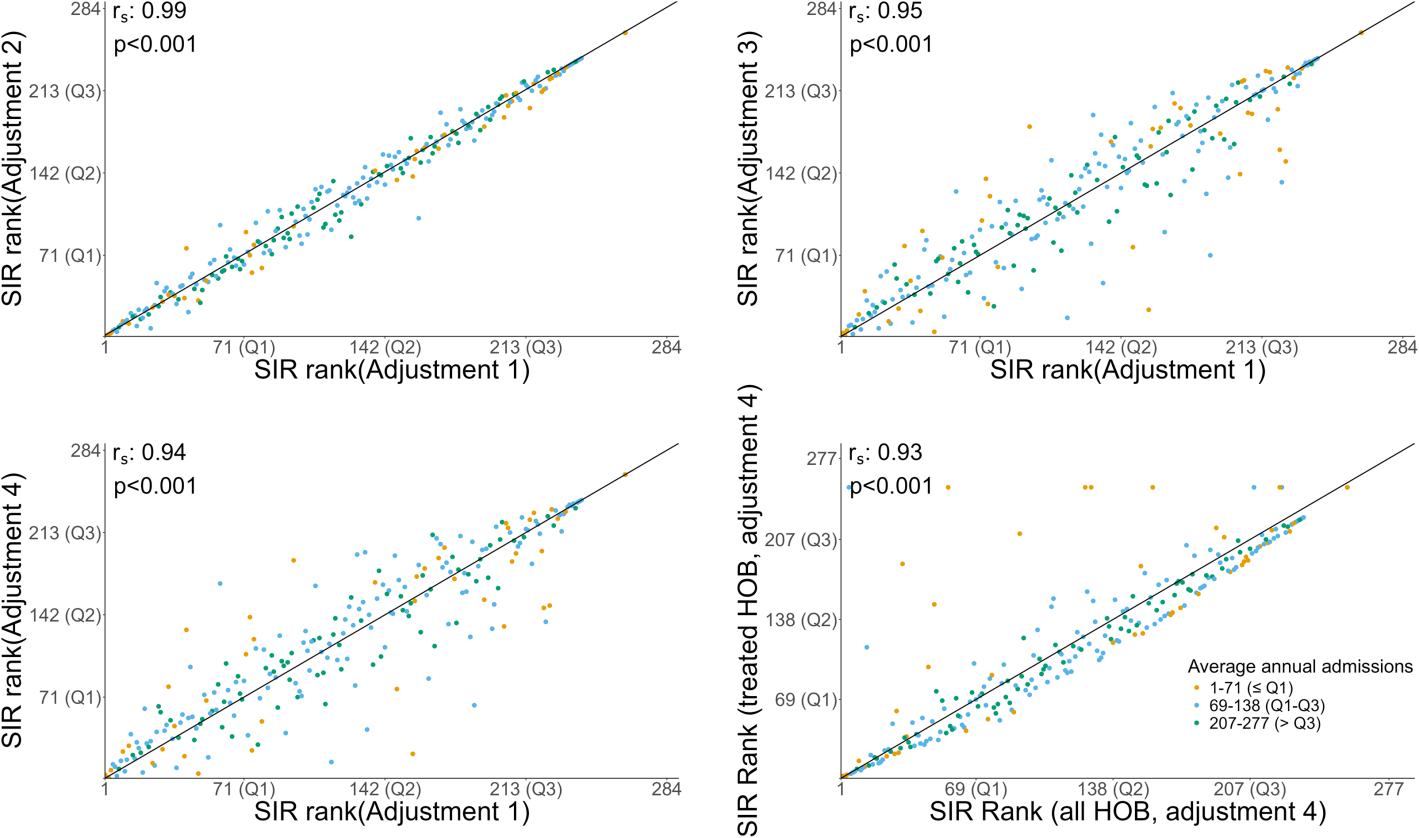
Scatterplots, with Spearman correlation coefficients, of HOB Standardized Infection Ratio (SIR) rank (ordered smallest to largest) derived from adjusted SIR model 1 compared to adjusted SIR model 2–4 (Panels A through C) for 284 neonatal intensive care units (NICUs) in the analysis. Risk adjustments include: birthweight (model 1), birthweight and postnatal age (model 2), birthweight and NICU complexity (model 3), and birthweight, postnatal age, and NICU complexity (model 4). Panel D displays adjusted SIR model 4 based on all HOB to the corresponding SIR rank using only non-commensal and treated commensal HOB events based on 277 NICUs that provided antibiotic data.

Sites were ranked from smallest to largest according to the unadjusted HOB rate, and these rankings were compared to rankings based on the 4 HOB SIR models (Figure 2). Fifty-five (19.4%) of the 284 NICUs moved out of or into the worst-performing category (ie the fourth quartile of HOB rate or SIR ranks) after any risk adjustment (Figure 3). The same sites did not always move up or down with each adjustment. As compared to the performance ranking using the unadjusted HOB rate, 16 (22.5%) of 71 NICUs moved out of the fourth quartile after adjusting for birthweight (model 1) (Table 1). When compared to SIR rank adjusted for birthweight (model 1), three (4.2%) NICUs moved out of the worst-performing group after birthweight and postnatal age adjustment (model 2), 13 (18.3%) after birthweight and NICU complexity adjustment (model 3), and 13 (18.3%) after birthweight, postnatal age, and NICU complexity adjustment (model 4) (Table 1). The 185 NICUs that remained in the first-third quartiles (better-performing) regardless of adjustment had a median unadjusted HOB rate of 0.5 (IQR: 0.1–0.8) compared to a rate of 1.7 (IQR: 1.5–2.2) in the 44 NICUs that remained in the fourth quartile (worst-performing) despite adjustment (Table 1). The median percentage of infants born <1500 g among sites that remained in the first-third quartiles was 8.7% (IQR: 4.8–12.6) and was 11.3% (IQR: 9.0–15.1) among sites that remained in the fourth quartile.

**Table 1.** Characteristics of NICUs included in the analysis, grouped by change in standardized infection ratio (SIR) rank after changes in the risk adjustment strategy

	N	Median (IQR)^a^ HOB^b^ Rate	Median (IQR) of Mean Annual Admissions	Median (IQR) Percentage Admitted <1500 g	Median (IQR) Percentage Outborn	Median (IQR) Percentage Died	Median (IQR) Site Central line Intensity	Median (IQR) Percentage Admitted after 42 Days
Sites with unchanged Quartiles after all Adjustments								
Remains in First-Third Quartile	185	0.5 (0.1, 0.8)	286 (181.4, 496.8)	8.7% (4.8, 12.6)	7.4% (3.8, 15.8)	0.5% (0.01, 12.0)	6.7% (3.7, 11.7)	0.0% (0.0, 0.0)
Remains in Fourth Quartile (Worst-Performing)	44	1.7 (1.5, 2.2)	396.8 (246.0, 674.0)	11.3% (9.0, 15.1)	9.4% (3.0, 21.5)	1.9% (1.0, 2.7)	13.9% (9.2, 19.2)	0.1% (0.1, 0.4)
Sites that left Fourth Quartile after SIR adjusted for birthweight (model 1) compared to unadjusted ranking								
Birthweight adjusted (model 1)	16	1.2 (1.2, 1.4)	683 (599.5, 864.0)	17.1% (15.8, 19.2)	13.2% (5.1, 22.0)	2.2% (1.8, 3.1)	17.9% (14.1, 19.5)	0.2% (0.1, 0.5)
Sites that left fourth quartile after additional SIR adjustment (model 2–4) compared to birthweight adjusted only (model 1)								
Birthweight and postnatal age adjusted (model 2)	3	1.4 (1.3, 1.5)	204 (192.2, 274.2)	10.9% (10.7. 14.0)	15.2% (8.8, 22.0)	1.7% (1.6, 1.8)	10.7% (9.0, 15.6)	0.0 (0.0, 0.0)
Birthweight and hospital complexity adjusted SIR (model 3)	13	1.4 (1.2, 1.7)	430 (262.6, 929.4)	10.7% (9.4, 16.4)	15.2% (12.4, 20.7)	1.5% (1.1, 2.4)	11.6% (10.7, 15.9)	0.1% (0.0, 0.3)
Birthweight, postnatal age and hospital complexity adjusted (model 3)	13	1.4 (1.2, 1.7)	382.6 (262.8, 929.4)	10.7% (9.4, 14.6)	13.5% (11.8, 19.0)	1.5% (0.1, 2.0)	11.5% (8.3, 14.6)	0.1% (0.0, 0.3)

a First and third interquartile range.

b Hospital-onset bacteremia.

Birthweight-adjusted (model 1) HOB SIR ranks correlated closely with birthweight and postnatal age-adjusted HOB SIR ranks (model 2) (Spearman correlation coefficient: 0.99, *P* < .001), birthweight and NICU complexity-adjusted HOB SIR ranks (model 3) (Spearman correlation coefficient 0.95, *P* < .001), and birthweight, postnatal age, and NICU complexity-adjusted HOB SIR ranks (model 4) (Spearman correlation coefficient 0.94, *P* < .001) (Figure 3). Furthermore, birthweight, postnatal age, and NICU complexity-adjusted HOB SIR ranks (model 4) using all HOB events were highly correlated with the computed HOB SIR ranks when only non-commensal and treated commensal cultures were included in the same model (Spearman correlation coefficient 0.93, *P* < .001) (Figure 3).

## Discussion

In this study of 284 NICUs, we provide the first exploration of risk adjustment models for NICU HOB rates and SIR calculations. The CDC plans to implement HOB as an electronically derived HAI measure. We demonstrated that infant and unit-level risk adjustment impact NICU HOB ranking and should be implemented in the future. On average, the sites that moved from the fourth (worst-performing) quartile into a better-performing quartile after adjustment were larger NICUs with a higher proportion of infants born <1500 g. Additional research is needed to optimize risk adjustment, and future models may include birthweight, postnatal age, and NICU complexity given that these patient and unit-level characteristics contributed to a change in site SIR rank in this large cohort.

Birthweight has consistently been an important risk factor for infant bacteremia and fungemia, and our recent analysis of this cohort demonstrated that infant birthweight has a strong, independent association with HOB.^9,19,23^ After adjusting for birthweight (model 1), 16 (22.5%) sites in the fourth quartile moved into a better-performing quartile. Currently, NHSN uses birthweight in addition to other unit-level variables to adjust for CLABSI, and our results demonstrate that birthweight should be included in future HOB risk adjustment. We previously showed that postnatal age is an important risk factor for infant HOB; however, the effect of postnatal age differs among birthweight groups.^19^ As compared to birthweight-adjusted SIR (model 1), only 3 (4.2%) sites left the fourth quartile after adding postnatal age to the model in addition to birthweight (model 2). This is likely due to the small number of infants born ≥1500 g who were admitted to NICUs for more than 42 days. Postnatal age adjustment may have a larger impact on HOB SIR in a nationally representative cohort that includes a greater number of NICUs with medically complex infants born ≥1500 g who have prolonged ICU admissions. Such infants are more likely to have major congenital anomalies that require prolonged admission and have different risks for infection as compared to preterm infants.

Adjusting for patient- and unit-level complexity can improve the accuracy of healthcare-associated infection models.^16,24,25^ Accurate comparison of units is essential for benchmarking and to drive performance improvement. As compared to birthweight-adjusted SIR (model 1), 13 (18.3%) sites left the fourth quartile after adding NICU complexity (model 3) or NICU complexity and postnatal age (model 4) to a model adjusting for birthweight alone. To contextualize what an 18.3% change would mean for nationwide data, 1,023 NICUs reported CLABSI data to NHSN in 2022.^26^ If a similar number of hospitals reported HOB, then 256 NICUs would compromise fourth quartile for HOB ranking, and approximately 46 NICUs (∼4.6% of all NICUs) would leave this quartile if HOB were adjusted for NICU complexity in addition to birthweight. Therefore, including both unit- and individual-level characteristics would impact HOB performance comparisons and benchmarking. Although adjusting for NICU complexity resulted in additional sites leaving the fourth quartile, the NICUs that remained in the fourth quartile regardless of adjustment had higher mean annual admissions, a higher proportion of infants born <1500 g, and a higher percentage of infants who died as compared to those that remained in the first-third quartiles. This suggests that our models adjusting for NICU complexity had residual unmeasured confounding, and further studies are needed in order to assess the most accurate and fair methods of adjusting for HOB risk.

Adult HOB analyses have excluded commensal organisms.^16^ We included commensal organisms because there is no consensus definition of a contaminated blood culture in the NICU, and commensal organisms, such as coagulase-negative staphylococci, can cause invasive infection among infants.^27,28^ In our cohort, SIR rank based upon all HOB events correlated closely with SIR rank based upon non-commensal and treated commensals. Undoubtedly, some of these commensals represent contamination events rather than invasive infections. Future research should focus on developing novel definitions and prediction models for identifying contaminants within this population.

Other studies have approached HOB adjustment differently than our analysis. Prior research has tested more variables and utilized goodness of fit testing to select models.^16,25^ We created models based upon *a priori*-selected clinical and unit-level variables that have been shown to be risk factors for HOB and would be readily available within a national database, respectively. This method was utilized because our goal was to demonstrate the impact of risk adjustment on SIR rank.

This study had limitations. We did not have unit-level variables that may improve risk adjustment, such as the American Academy of Pediatrics NICU level, Vermont Oxford Network NICU type, or data regarding center infection prevention practices.^29,30^ Future studies with more granular, unit-level data may find that alternative, unit-level variables have an even greater impact on risk-adjusted HOB SIR ranking than those explored in this analysis. We included patient-level variables that are established risk factors for HOB, such as birthweight and postnatal age; however, we were unable to adjust for additional markers of patient complexity, such as surgeries and procedures. We did not have complete data from all sites, and 19 sites were excluded due to having missing culture data. Our risk adjustment strategies also intentionally excluded small units with an expected HOB event of less than 1, which reflects current NHSN practice. Despite these limitations, this study provides the first description of NICU HOB risk adjustment using a large retrospective, multicenter cohort and is important to inform future risk adjustment decision-making.

Adjusting for patient and unit-level complexity improves HAI benchmarking and unit comparison, which are needed to inform unit healthcare quality priorities and resources.^12,24,25^ Future studies should continue to evaluate important risk adjustment variables for NICU HOB. Within our cohort, birthweight adjustment caused 22.5% of the sites in the worst-performing quartile to move into a better-performing quartile, indicating that birthweight should continue to be used for risk adjustment. Further adjustment for NICU complexity in addition to birthweight led to 18.3% of the sites in the worst-performing quartile to move into a better-performing quartile as compared to a model adjusting for only birthweight. Nevertheless, the sites that remained in the worst-performing quartile after all adjustments were generally larger NICUs with a higher proportion of infants born <1500 g. Therefore, further risk adjustment may be required to account for unit-level complexity. Rigorous risk adjustment of NICU HOB will allow for fair benchmarking of unit HOB rates and guide performance improvement.
